# Supplementary material for: Research on the cooperative mechanism of government and enterprise for basin ecological compensation based on differential game
Source: PLoS One. 2021 Jul 23;16(7):e0254411. doi: 10.1371/journal.pone.0254411 (PMC8302252; doi:10.1371/journal.pone.0254411)
Supplement: Attachment — Submitted filename: Comments_R1.pdf [file pone.0254411.s002.pdf]

Referee Report

Title: Research on the Cooperative Mechanism of Government and Enterprise for Basin Ecological Compensation Based on Differential Game
Authors: Sun Hao, Gao Guang-kuo, Li Zong-huo

The authors have made great improvements to the manuscript. The scope of the study has been broadened some by including a detailed literature review. In order for publication in PLoS One, I still have some changes to recommend here.

1. Although authors have made most corrections for the compensation mechanism solutions, there are still some errors in the terms of writing. For example, in Eq. (2) and (24), the advertising effect coefficient is "α", while in the other expressions, this parameter is replaced by "a". I recommend authors should keep it consistent. In Eq. (22), I believe "2A-A" should be "2A-B". Besides, I cannot get the results shown in Eqs. (29-30) when substituting the expressions (25), (27) and (28) into (24) and (26) except the numerator of the "L" should be "$\pi_1 a$" or "$\pi_1 \alpha$" rather than "$\pi_1 \mu$". When substituting Eq. (42) into Eq. (41), I found it should be "$+$" instead of "-" in the first term of Eq. (44). In Eq. (47), the term "$1/\rho$" in Eq. (46) miss out. I cannot obtain the Eq. (48), since the factor α in front of π_2 in Eq. (22) is lost. I highly recommend authors to double check all the expressions again.

2. I would suggest that the authors should address the captions of Figs. 4-6 in detail. The captions should include the interpretation of x-axis and y-axis, the use of parameters, and the implication of lines in each figure, etc. In addition, the text size of figures is a little bit small. Therefore, I recommend that each figure should be taken separately for clear visualization and analysis.

3. The authors have chosen only one set of parameters for the calculation analysis. I am curious to see how the results would change if other parameters were chosen.
