# Supplementary material for: Research on the cooperative mechanism of government and enterprise for basin ecological compensation based on differential game
Source: PLoS One. 2021 Jul 23;16(7):e0254411. doi: 10.1371/journal.pone.0254411 (PMC8302252; doi:10.1371/journal.pone.0254411)
Supplement: Attachment — Submitted filename: Comments_R2.pdf [file pone.0254411.s004.pdf]

Referee Report

Title: Research on the Cooperative Mechanism of Government and Enterprise for Basin Ecological Compensation Based on Differential Game
Authors: Sun Hao, Gao Guang-kuo, Li Zong-huo

I believe the manuscript has been improved a lot. However I would like to recommend some changes.

1. There are still some mistakes in expressions. For example, in Eq. (23), it should be $\lambda_2\pi_1\theta$ instead of $\lambda_2\pi_2\theta$. I believe that in Eq. (48), the results of $S_2^{D^*} - S_2^{N^*}$ should be simplified as $\frac{2A-B}{2k_2}$ with expressions A and B. I am confused with the expression of Eq. (49) since there is no formula "b" defined in the manuscript. I found the $(\rho+\delta)^2$ is missed out in the denominator of Eq. (51). In addition, there are still formulas in which the parameter of advertising effect coefficient is written as "a" rather than "α", such as in Eq. (11), (14), (18), (34) and (46). Although most of them appear in the comparative analysis where the mistakes would not make a large difference, please make sure to do a more detailed check of all equations before the submission.

With the above point properly addressed, I would recommend the paper is ready for publication in PLOS ONE.
