# Supplementary material for: Research on the cooperative mechanism of government and enterprise for basin ecological compensation based on differential game
Source: PLoS One. 2021 Jul 23;16(7):e0254411. doi: 10.1371/journal.pone.0254411 (PMC8302252; doi:10.1371/journal.pone.0254411)
Supplement: Attachment — Submitted filename: 1Response to Reviewers.doc [file pone.0254411.s003.doc]

Dear Editor and Reviewers:

We thank you very much for giving us an opportunity to revise our manuscript and appreciate your positive and constructive comments very much. We have studied comments carefully and made corrections which, we hope, have improved the quality of the paper to your satisfaction. The revisions have been highlighted across the text. Our responses to your valuable comments and the main corrections in the paper are as follows:

**Comment 1:** Although authors have made most corrections for the compensation mechanism solutions, there are still some errors in the terms of writing. For example, in Eq. (2) and (24), the advertising effect coefficient is “α”, while in the other expressions, this parameter is replaced by “a”. I recommend authors should keep it consistent. In Eq. (22), I believe “2A-A” should be “2A-B”. Besides, I cannot get the results shown in Eqs. (29-30) when substituting the expressions (25), (27) and (28) into (24) and (26) except the numerator of the “L” should be “π1a” or “π1α” rather than “π1µ”. When substituting Eq. (42) into Eq. (41), I found it should be “+” instead of “-” in the first term of Eq. (44). In Eq. (47), the term “1/ρ” in Eq. (46) miss out. I cannot obtain the Eq. (48), since the factor α in front of π2 in Eq. (22) is lost. I highly recommend authors to double check all the expressions again.

**Response:** Thank you for your comments. Following your comment, we have carefully checked all the expressions in the paper and corrected the errors. The revisions have been highlighted across the text.

**Comment 2:** I would suggest that the authors should address the captions of Figs. 4-6 in detail. The captions should include the interpretation of x-axis and y-axis, the use of parameters, and the implication of lines in each figure, etc. In addition, the text size of figures is a little bit small. Therefore, I recommend that each figure should be taken separately for clear visualization and analysis.

**Response:** Thanks for your valuable comment. Following your comment, we explained the meaning of the X-axis and Y-axis and the related parameters in the figure. In addition, we analyze each figure separately and explain the meaning of the lines in each figure.

**Comment 3:** The authors have chosen only one set of parameters for the calculation analysis. I am curious to see how the results would change if other parameters

were chosen.

**Response:** Thank you for your valuable suggestion. Following your comment, we have added the sensitivity analysis table of related parameters, and the influence of the change of related parameters on the results is analyzed. We believe that these changes are in line with your requirements.

Special thanks to you for your careful review and good comments, we hope that our modifications can meet your requirements.
