# Supplementary material for: Research on the cooperative mechanism of government and enterprise for basin ecological compensation based on differential game
Source: PLoS One. 2021 Jul 23;16(7):e0254411. doi: 10.1371/journal.pone.0254411 (PMC8302252; doi:10.1371/journal.pone.0254411)
Supplement: Attachment — Submitted filename: 623Response to Reviewers.doc [file pone.0254411.s005.doc]

Dear Editor and Reviewers:

We thank you very much for your comments. We have studied comments carefully and made corrections which, we hope, have improved the quality of the paper to your satisfaction. The revisions have been highlighted across the text. Our responses to your valuable comments and the main corrections in the paper are as follows:

**Comment 1:** There are still some mistakes in expressions. For example, in Eq. (23), it should be$\lambda_{2}\pi_{1}\theta$instead of$\lambda_{2}\pi_{2}\theta$. I believe that in Eq. (48), the results of $S_{2}^{D*}-S_{2}^{N*}$should be simplified as$\frac{2A-B}{2k_{2}}$with expressions A and B. I am confused with the expression of Eq. (49) since there is no formula “*b*” defined in the manuscript. I found the (*ρ*+*δ*)^2^ is missed out in the denominator of Eq. (51). In addition, there are still formulas in which the parameter of advertising effect coefficient is written as “*a*” rather than “*α*”, such as in Eq. (11), (14), (18), (34) and (46). Although most of them appear in the comparative analysis where the mistakes would not make a large difference, please make sure to do a more detailed check of all equations before the submission.

**Response:** Thank you for your comments. Following your comment, we have carefully checked all the expressions in the paper and corrected the errors. The revisions have been highlighted across the text.

Special thanks to you for your careful review and good comments, we hope that our modifications can meet your requirements.
